# Cathepsin L promotes chemresistance to neuroblastoma by modulating serglycin

**DOI:** 10.3389/fphar.2022.920022

**Published:** 2022-08-26

**Authors:** Xiaohuan Du, Leyun Ding, Shungen Huang, Fang Li, Yinghui Yan, Ruze Tang, Xinyuan Ding, Zengyan Zhu, Wenjuan Wang

**Affiliations:** ^1^ Department of Pharmacy, Children’s Hospital of Soochow University, Suzhou, China; ^2^ Department of Pharmacy, The Affiliated Suzhou Hospital of Nanjing Medical University, Suzhou, China; ^3^ Department of Oncology, Children’s Hospital of Soochow University, Suzhou, China

**Keywords:** chemoresistance, neuroblastoma, Cathepsin L, autophagy, apoptosis

## Abstract

Cathepsin L (CTSL), a lysosomal acid cysteine protease, is found to play a critical role in chemosencitivity and tumor progression. However, the potential roles and molecular mechanisms of CTSL in chemoresistance in neuroblastoma (NB) are still unclear. In this study, the correlation between clinical characteristics, survival and CTSL expression were assessed in Versteeg dataset. The chemoresistant to cisplatin or doxorubicin was detected using CCK-8 assay. Western blot was employed to detect the expression of CTSL, multi-drug resistance proteins, autophagy-related proteins and apoptosis-related proteins in NB cells while knocking down CTSL. Lysosome staining was analyzed to access the expression levels of lysosomes in NB cells. The expression of apoptosis markers was analyzed with immunofluorescence. Various datasets were analyzed to find the potential protein related to CTSL. In addition, a subcutaneous tumor xenografts model in M-NSG mice was used to assess tumor response to CTSL inhibition *in vivo*. Based on the validation dataset (Versteeg), we confirmed that CTSL served as a prognostic marker for poor clinical outcome in NB patients. We further found that the expression level of CTSL was higher in SK-N-BE (2) cells than in IMR-32 cells. Knocking down CTSL reversed the chemoresistance in SK-N-BE (2) cells. Furthermore, combination of CTSL inhibition and chemotherapy potently blocked tumor growth *in vivo*. Mechanistically, CTSL promoted chemoresistance in NB cells by up-regulating multi-drug resistance protein ABCB1 and ABCG2, inhibiting the autophagy level and cell apoptpsis. Furthermore, we observed six datasets and found that Serglycin (SRGN) expression was positively associated with CTSL expresssion. CTSL could mediate chemoresistance by up-regulating SRGN expression in NB cells and SRGN expression was positively correlated with poor prognosis of NB patients. Taken together, our findings indicate that the CTSL promotes chemoresistance to cisplatin and doxorubicin by up-regulating the expression of multi-drug resistance proteins and inhibiting the autophagy level and cell apoptosis in NB cells. Thus, CTSL may be a therapeutic target for overcoming chemoresistant to cisplatin and doxorubicin in NB patients.

## Introduction

Neuroblastoma (NB) is the most common extracranial solid tumor in children. NB accounts for 10% of all childhood cancers and also 15% of pediatric cancer deaths (Tolbert and Matthay, 2018). Main available treatment strategy for NB patients is chemotherapy followed by surgical resection. Cisplatin (DDP) and doxorubicin (ADM) are common chemotherapeutics for NB (Jemaà et al., 2020). However, the clinical efficacy of these drugs is limited by chemoresistance, which is the main cause of the treatment failure in NB patients (Rodrigo et al., 2021). Thus, it is important to research the molecular mechanisms of chemoresistance in NB and find the potential targets to overcome it.

Various molecular mechanisms have been implicated in chemoresistance. The ATP-binding cassette transporter (ABC) family of transmembrane proteins is associated with chemoresistance by promoting drug efflux. Among these, ABC transporter B1 (ABCB1/MDR1/P-glycoprotein), ABC transporter C1 (ABCC1/MRP1) and breast cancer resistance protein (ABCG2/BCRP) are closely related to poor platinum sensitivity (Domenichini et al., 2019). Resistance to apoptosis can also cause chemoresistance. Mutations, amplifications and overexpression of the genes encoding the anti-apoptotic BCL-2 family members and inhibitor of apoptosis proteins (IAPs) have been reported associated with chemoresistance of cancers. Autophagy is another mechanism of chemoresistance by promoting cancer cell survival during metabolic stresses induced by anticancer agents. Besides, alterations in drug metabolism, DNA damage repair, epigenetic changes, mutation of drug targets and the influence of tumor microenvironment may also contribute to chemoresistance of cancers (Holohan et al., 2013).

The ability of cancer cells to maintain an internal stasis is a critical characteristic of a neoplasm (Li et al., 2017). The important role of lysosomes in cellular stasis has been identified in many studies (Yang and Wang, 2021). Lysosome is connected to chemoresistance, cellular adaptation, immune response and cell death (Hraběta et al., 2020). Lysosomes have more than 60 hydrolytic enzymes, including proteases, lipases (Davidson and Vander Heiden, 2017). Tumor stasis is a multidimensional process that is adjusted by cellular proteins, including cathepsin family of proteases, protein-protein interactions, alternative splicing and expression of miRNAs (Mijanović et al., 2019). Cathepsins play important roles in malignant tumors. Cathepsin L (CTSL) is a cysteine protease which has been reported linked to tumor occurrence, development, and metastasis (Sudhan and Siemann, 2015). CTSL up-regulation has been identified in many human malignancies including gastric (Pan et al., 2020) and lung (Wang et al., 2018) cancers. Significantly, CTSL has important roles in regulating cancer chemoresistance (Zhao et al., 2019). Our previous study found that CTSL up-regulation-induced EMT phenotype was associated with the acquisition of DDP or paclitaxel resistance in A549 cells (Han et al., 2016). However, the roles and the mechanisms of CTSL in NB chemoresistance are still unclear which need to be studied further.

In the present study, we demonstrated that CTSL was a regulator of poor DDP and ADM sensitivity in NB cells, and the regulation of chemoresistance by CTSL was mediated through its effects on ABC proteins, autophagy and cell apoptosis. These findings indicate that CTSL may represent a novel therapeutic target to overcome poor DDP and ADM sensitivity in NB patients.

## Materials and methods

### Validation of human datasets

Tissue array analysis results of NB patient tumor samples were obtained from the R2 Genomics Analysis and Visualization Platform (http://r2.amc.nl) using the following publicly available dataset: Versteeg (GEO: GSE16476) (Zhang H. et al., 2020), which included comprehensive information on the relevant clinical and prognostic factors selected for analysis. For Kaplan-Meier analysis, the best *p* value and corresponding cutoff value was selected according to the R2 Genomics Analysis and Visualization Platform.

Six datasets (including Tumor Neuroblastoma—Seeger—117—DCHIP, Westermann—144—tpm, Fischer—223—custom, Jagannathan—100—custom, Maris—101—custom and SEQC—498—custom) were observed at the same time and different colors represented different datasets in the box plot after standardization, According to −0.6 < R < 0.6 and *p* < 0.001 and at the top 20 of the datasets, the eligible common genes in six databases could be screened out. Then the Spearman correlation analysis between the selected gene and CTSL was performed.

### Cell lines and culture

The human NB cell lines, SK-N-BE (2) and IMR-32 were obtained from the Type Culture Collection of the Chinese Academy of Sciences, Shanghai, China. SK-N-BE (2) and IMR-32 cells were cultured in a 1:1 mixture of MEM and DMEM/F-12 with 10% fetal bovine serum, 1% Penicillin-Streptomycin. These cells were placed in an incubator with 5% CO₂ at 37°C and passaged every 72 h.

### CCK-8 assay

Cell Counting Kit-8 (CCK-8) assay was used to measure the viability and proliferation of cells. SK-N-BE (2) and IMR-32 cells in logarithmic growth phase, with the appropriate concentration of 5 × 10^4^/ml, were inoculated into 96-well culture plates with 100 μl/well, cultured overnight in an incubator with 5% CO₂ at 37°C and treated the next day. After pretreatment with different concentrations of DDP (HY-17394, MedChemExpress, Shanghai) or ADM (HY-15142A, MedChemExpress, Shanghai) for 24 h, 10 μl CCK-8 solution was added to each well and incubated for 4 h at 37°C. The optical density was measured at 450 nm. All assays were performed in triplicate.

### siRNA transfection

CTSL siRNA, SRGN siRNA, and negative control siRNA were purchased from Sangon Biotech (Shanghai, China). For transfection, siRNA was mixed with Lipofectamine^®^ 3000 Reagent (Invitrogen) and then transfected into SK-N-BE (2) or IMR-32 cells. After 6 h, the supernatant was replaced with fresh medium containing 10% FBS and cultured for another 24 h. Three siRNA sequences were used for transfection (Table 1).

**TABLE 1:** Sequences for CTSL siRNA and SRGN siRNA.

si-RNA	Base sequence	
Negative control (NC)	sense	5′-UUC​UCC​GAA​CGU​GUC​ACG​UTT-3′
	antisense	5′-ACG​UGA​CAC​GUU​CGG​AGA​ATT-3′
CTSL-homo-450	sense	5′-GCG​AUG​CAC​AAC​AGA​UUA​UTT-3′
	antisense	5′-AUA​AUC​UGU​UGU​GCA​UCG​CTT-3′
CTSL-homo-994	sense	5′-CCA​AGU​AUU​CUG​UUG​CUA​ATT-3′
	antisense	5′-UUA​GCA​ACA​GAA​UAC​UUG​GTT-3′
CTSL-homo-1112	sense	5′-CCU​UCC​UGU​UCU​AUA​AAG​ATT-3′
	antisense	5′-UCU​UUA​UAG​AAC​AGG​AAG​GTT-3′
SRGN-homo-123	sense	5′-CCU​CAG​UUC​AAG​GUU​AUC​CUA​TT-3′
	antisense	5′-UAG​GAU​AAC​CUU​GAA​CUG​AGG​TT-3′
SRGN-homo-295	sense	5′-CCA​GGA​CUU​GAA​UCG​UAU​CUU​TT-3′
	antisense	5′-AAG​AUA​CGA​UUC​AAG​UCC​UGG​TT-3′
SRGN-homo-502	sense	5′-ACA​UGG​AUU​AGA​AGA​GGA​UUU​TT-3′
	antisense	5′-AAA​UCC​UCU​UCU​AAU​CCA​UGU​TT-3′

### Cathepsin L overexpressing cell line establishment

A lentivirus carrying CTSL gene was constructed by GeneChem (Shanghai, China). IMR-32 cells were seeded in 6-well plate and then infected with the lentivirus according to protocols as recommended by the manufacturer. After 16 h, the medium was replaced with complete medium. In order to obtain a stable CTSL overexpressing cell line, the lentivirus infected cells were selected by incubation with complete medium of 2 μg/ml puromycin. The expression of CTSL in IMR-32 cell lines stably infected with a lentivirus was examined by Western blot.

### Western blot analysis

Detailed procedure was as described in a previous study (Wang et al., 2019). Primary anti-human antibody against SRGN was purchased from Santa Cruz Biotechnology. CTSL, ABCB1, ABCG2, LC3, Bax, Bcl-2, and GAPDH primary anti-human antibodies were all purchased from Cell Signaling Technology.

### Real-time quantitative PCR

Detailed procedure for these steps has been previously reported (Gu et al., 2018). The primer sequences employed for the PCR analysis were listed in Table 2. All primers were synthesized by Sangon Biotech (Shanghai, China).

**TABLE 2:** Primers for CTSL and SRGN.

Gene	Primer	Primer sequence
CTSL	forward	5′-AAA​CTG​GGA​GGC​TTA​TCT​CAC​T-3′
	reverse	5′-GCA​TAA​TCC​ATT​AGG​CCA​CCA​T-3′
SRGN	forward	5′-GGA​CTA​CTC​TGG​ATC​AGG​CTT-3′
	reverse	5′-CAA​GAG​ACC​TAA​GGT​TGT​CAT​GG-3′

### Immunofluorescence

Cells were cultured on glass coverslips and then fixed with 4% paraformaldehyde. After a PBS wash, the cells were permeabilized employing 0.1% Triton X-100, incubated in a blocking solution (PBS with 3% bovine serum albumin), then further incubated overnight at 4°C with the primary antibody to Bcl-2, SRGN (Santa Cruz Biotechnology) and CTSL (Abcam). The fluorescent conjugated secondary antibodies were Alexa Fluor 488 and Alexa Fluor 594 (Invitrogen, Carlsbad, California, United States), and 4′,6-diamidino-2-phenylindole (DAPI) (Sigma Aldrich, St Louis, MO) was employed as a nuclear counterstain for 10 min. The coverslips were finally mounted onto slides with fluorescent mounting medium and instantly observed by confocal microscopy.

### Lysosome staining

After removing the cell culture solution, the lysosome green fluorescent probe staining solution (Beyotime, Nantong, China) was added, and the lysosome green fluorescent probe solution and the cell culture solution were mixed at a ratio of 1/20,000, and then incubated with cells in an incubator with 5% CO₂ at 37°C for 60 min. The staining solution was discarded and new cell culture solution was added. Finally the cells were observed under the laser confocal microscope.

### Apoptotic assays

Apoptotic cell death was assessed by flow cytometry using the AnnexinV-fluorescein isothiocyanate (FITC)/propidium iodide (PI) Apoptosis Detection Kit (KeyGEN). Cells were harvested and resuspended in binding buffer, then labeled with Annexin V-FITC/PI reagent for 15 min in the dark at room temperature. For each analysis, a minimum of 20,000 cells per sample were analyzed using a Beckman Coulter FACS machine. Results were analyzed and calculated by FlowJo V.10 software; the percentage of apoptosis was obtained from the sum of (Annexin V-FITC+/PI−) and (Annexin V-FITC+/PI+) cells.

### Animal experiments

All mice experiments were conducted in accordance with the humane treatment of animals under institutional guidelines approved by the Ethical Committee of Children’s Hospital of Soochow University. The mice were housed in individually ventilated cages in the Animal Laboratory of the Children’s Hospital of Soochow University. Six-week-old male M-NSG (NOD-Prkdc^scid^Il2rg^em1^/Smoc) mice (Shanghai Model Organisms, Shanghai) were used in the study. Subcutaneous tumor transplantation was conducted using the SK-N-BE (2) cells. Cells (*n* = 1 × 10^7^) were resuspended in 100 μl PBS and implanted into the right flank of nude mice under sterile conditions. After the formation of palpable tumors (tumor volume reached 100 mm^3^), mice were randomized into four groups (5 mice per group): control group (saline, i.p.), Z-FY-CHO (a specific CTSL inhibitor, HY-128140, MedChemExpress, Shanghai) group (5 mg/kg, i.p.), ADM group (1 mg/kg, i.p.), Z-FY-CHO (5 mg/kg, i.p.) plus ADM (1 mg/kg, i.p.) group. Mice were injected with vehicle or with drugs three times weekly. The size of the tumor and the body weight of each mouse were measured as described previously (Wang et al., 2019). Mice were sacrificed on day 15, and tumor tissues were harvested.

### Statistical analysis

All measurement data were expressed as the mean ± S.D. at least three independent experiments were conducted. Differences in measured variables between the experimental and control groups were assessed by Student’s *t*-test. The chi-square (χ^2^) test or Fisher exact test was used to compare qualitative variables. Differences were considered statistically significant at *p* values of <0.05. All analyses were performed employing GraphPad Prism 5.0.

## Results

### High expression of cathepsin L was positively associated with poor prognosis of NB patients

**FIGURE 1:**
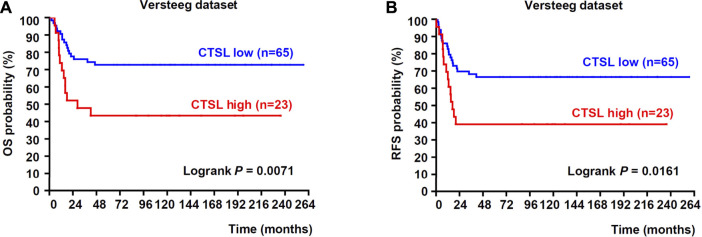
Upregulation of CTSL correlated with poor prognosis in patients with NB **(A)** Kaplan-Meier analysis of overall survival (OS) was determined according to CTSL expression in 88 NB samples from Versteeg dataset **(B)** Recurrence-free survival (RFS) was also determined according to CTSL expression from Versteeg dataset. Statistical analysis was carried out using a log-rank test.

To figure out the relationship between the expression of CTSL and prognosis of NB patients, we analyzed the public dataset Versteeg to examine the correlation between the mRNA expression of CTSL and the survival rates in NB. In the Versteeg dataset, patients with high CTSL mRNA levels (cut off: 615.00) showed significantly poor overall survival (OS) (22-years OS; *p* = 0.0071; Figure 1A) and poor recurrence-free survival (RFS) (22-years RFS; *p* = 0.0161; Figure 1B). Besides, increased CTSL expression was positively associated with advanced tumor stages, though there was no statistical significance (Supplementary Figure S1). In general, these results suggested that high expression of CTSL was positively correlated with poor prognosis of NB patients and CTSL might be a potential prognostic marker in NB.

### The expression of cathepsin L was poorly associated with ADM and DDP sensitivity in NB cells

**FIGURE 2:**
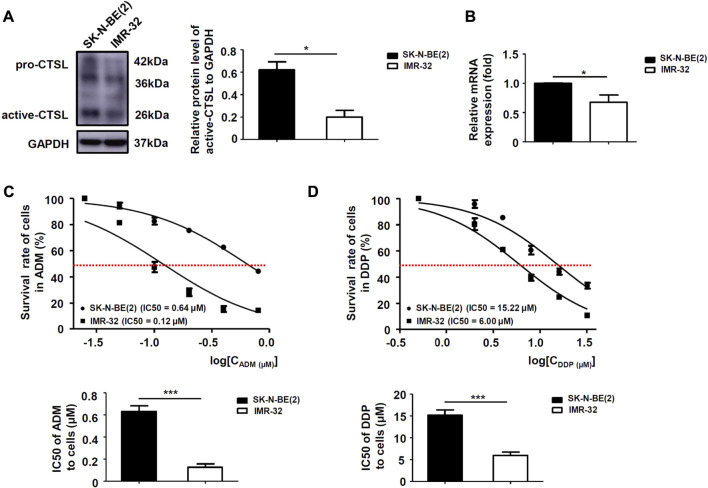
The expression of CTSL was associated with ADM and DDP sensitivity in NB cells **(A)** Western blot analysis and **(B)** real-time quantitative PCR analysis of CTSL levels in SK-N-BE (2) cells and IMR-32 cells. Cell viability curves for the two NB cell lines after ADM **(C)** and DDP **(D)** treatment were evaluated using the cell counting kit-8 assay (upper panel). The IC50 values were analyzed using the Mann-Whitney test (lower panel). *, *p* < 0.05, ***, *p* < 0.001.

To determine whether CTSL expression might be related to chemoresistance in NB cells, protein and mRNA levels of CTSL in two subtypes of NB cells were analyzed. Western blot and PCR analysis showed that the protein and mRNA levels of CTSL were low in IMR-32 cells and high in SK-N-BE (2) cells (Figures 2A,B). CCK-8 assay was performed to detect the 50% inhibitory concentration (IC50) of two cell lines in the treatment of a gradient concentration of ADM and DDP at 24 h. It turned out that the IC50 of SK-N-BE (2) cells in the treatment of ADM and DDP were higher than that of IMR-32 (*p* < 0.001; Figures 2C,D). These results suggested that SK-N-BE (2) cells were more chemoresistant to ADM and DDP than IMR-32 cells and CTSL expression was poorly associated with ADM and DDP sensitivity in NB cells.

### Cathepsin L down-regulated ADM and DDP sensitivity in NB cells

**FIGURE 3:**
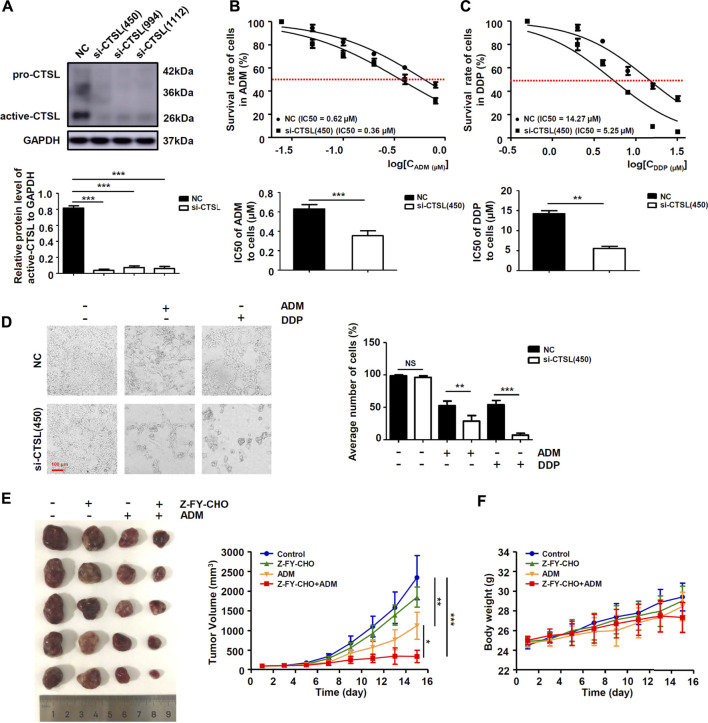
CTSL down-regulated ADM and DDP sensitivity in NB cells **(A)** Western blot showing CTSL expression in SK-N-BE (2) cells after CTSL silencing. Cell viability curves for SK-N-BE (2)/NC cells and SK-N-BE (2)/si-CTSL cells after ADM **(B)** and DDP **(C)** treatment were evaluated using the Cell Counting Kit-8 assay (upper panel). The IC50 values were analyzed using the Mann-Whitney test (lower panel) **(D)** Representative micrographs of two SK-N-BE (2)/NC cells and SK-N-BE (2)/si-CTSL cells after 50% lethal dose of ADM and DDP treatment (red bar: 100 μm) **(E)** and **(F)** Subcutaneous tumor xenograft models established using SK-N-BE (2) cells. Mice were injected with ADM or with Z-FY-CHO three times weekly. The tumor volumes were measured and the nude mice were weighed every 2 days until the mice were sacrificed. The curves were shown respectively. NS: not statistically significant, *, *p* < 0.05, **, *p* < 0.01, ***, *p* < 0.001.

We further researched whether chemoresistance was modulated by CTSL in NB cells. Western blot showed that all three selected si-RNAs could significantly down-regulate CTSL expression of SK-N-BE (2) cells, we chose si-CTSL (450) with the best interference effect to interfere the CTSL expression of SK-N-BE (2) cells (Figure 3A). CCK-8 assay was performed to detect the IC50 of SK-N-BE (2) cells in the treatment of a gradient concentration of ADM and DDP at 24 h in the reference of si-CTSL (450) and a specific CTSL inhibitor, Z-FY-CHO. We found that the IC50 of si-CTSL (450) group and Z-FY-CHO group under the action of ADM and DDP of various concentrations were lower than that of NC group and control group, which showed that the chemosensitivity of SK-N-BE (2) cells to ADM and DDP increased after CTSL inhibition (Figures 3B,C; Supplementary Figures S2A,B). Considering that CTSL may be closely related to the development of chemoresistance. A lentivirus carrying CTSL gene was constructed and infected into IMR-32 cells (Supplementary Figure S3A). Overexpression of CTSL rendered them resistant to ADM and DDP, as indicated by CCK-8 assay compared with LV-Vector cells, confirming the chemoresistant role of CTSL in NB cells (Supplementary Figure S3B,C). Then, the 50% lethal dose of ADM and DDP were selected to treat SK-N-BE (2) cells. The morphological changes of NC group and si-CTSL (450) group after drug treatment were observed. In NC group, the cells were inhibited to some extent, and the number of cells decreased after administration with ADM or DDP, but some cells still survived. In si-CTSL (450) group, the cell morphology changed from fusiform to round after administration with ADM or DDP, its lethality to cells also increased greatly (Figure 3D). We further investigated the effect of CTSL on chemoresistance of NB *in vivo*. The subcutaneous tumor xenograft model was established using SK-N-BE (2) cells, and then treated with Z-FY-CHO or ADM. As shown in Figure 3E, tumor volume was 2345.20 ± 561.75 mm^3^ in the control group, 1850.10 ± 255.30 mm^3^ in Z-FY-CHO group, 1124.80 ± 343.89 mm^3^ in ADM group, and 344.60 ± 156.03 mm^3^ in Z-FY-CHO plus ADM group. The average volumes of tumors were remarkably decreased in Z-FY-CHO plus ADM group compared to ADM group (*p* < 0.05), which was consistent with the results of experiments *in vitro*. The weights of mice were also recorded. As was shown, there was no significant difference among those groups in tumor weight (Figure 3F). The results of tumor xenograft experiment confirmed that CTSL inhibition down-regulated chemoresistance of NB.

### Cathepsin L induced chemoresistance to ADM and DDP in NB cells by up-regulating the expression of multi-drug resistance proteins and inhibiting the autophagy level.

**FIGURE 4:**
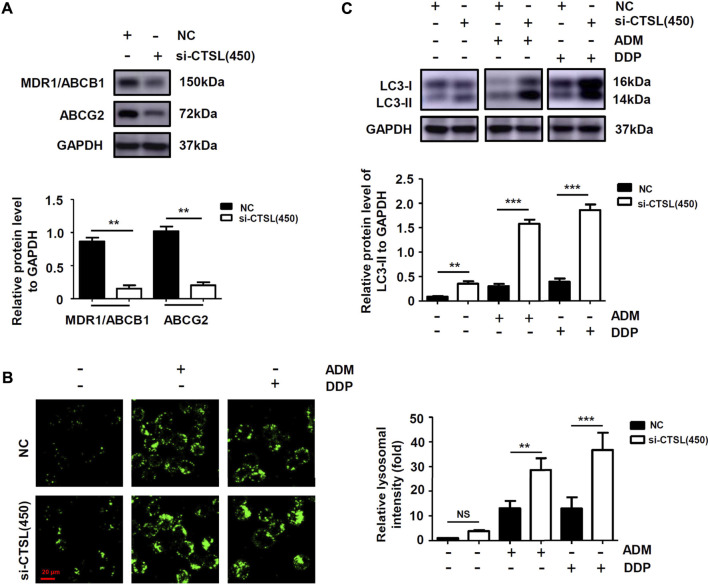
The expression of multi-drug resistance proteins and autophagy level of NB cells after knockdown of CTSL **(A)** Western blot showing multi-drug resistance proteins expression in SK-N-BE (2) cells after CTSL silencing **(B)** Lysosome staining assay showing the quantity and intracellular location of lysosome in SK-N-BE (2) cells with different treatments (red bar: 20 μm) **(C)** The expression of LC3-I and LC3-II in SK-N-BE (2) cells were analyzed using Western blot. NS: not statistically significant, **, *p* < 0.01, ***, *p* < 0.001.

To explore the mechanisms of chemoresistance in NB cells with high CTSL expression, Western blot was used to detect the expression level of ABCB1 and ABCG2 in NC group and si-CTSL group. It turned out that the expression levels of ABCB1 and ABCG2 were lower in si-CTSL group than that of NC group (Figure 4A; Supplementary Figures S4A,B). The results showed that knockdown of CTSL down-regulated the expression of multi-drug resistance proteins. Further, to figure out whether CTSL expression was associated with autophagy in NB cells, we observed the expression level of lysosomes in NC group and si-CTSL (450) group and found that the green fluorescent spots in si-CTSL group were more and brighter compared with NC group (Figure 4B). This finding indicated that the autophagy level of si-CTSL group may be higher than that of NC group. Furthermore, Western blot was employed to detect the expression of autophagy-related protein LC3-Ⅱ of NC group and si-CTSL group at the same concentration of ADM and DDP. It turned out that the expression of LC3-Ⅱ of si-CTSL group was higher than that of NC group (Figure 4C; Supplementary Figures S4C,D). These results suggested that CTSL down-regulated ADM and DDP sensitivity in NB cells by inhibiting the autophagy level. Thus, all these findings indicated that CTSL could induce chemoresistance to ADM and DDP in NB cells by up-regulating the expression of multi-drug resistance proteins and inhibiting the autophagy level.

### Cathepsin L induced chemoresistance to ADM and DDP in NB cells by inhibiting cell apoptosis

**FIGURE 5:**
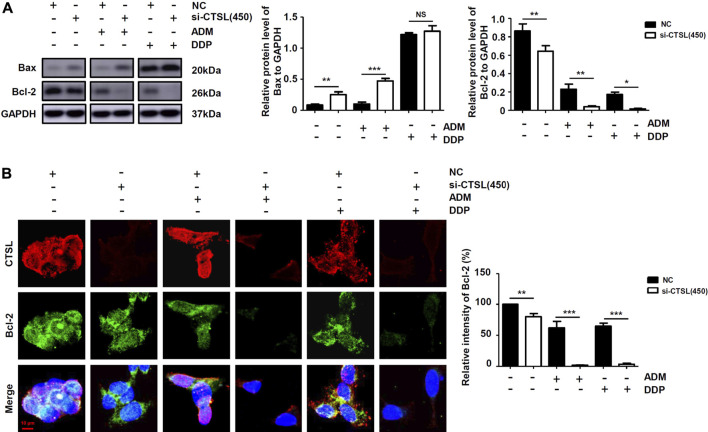
The apoptosis level of NB cells after knockdown of CTSL **(A)** Western blot showing apoptosis proteins expression in SK-N-BE (2) cells with different treatments **(B)** Immunofluorescence assay showing the expression and intracellular location of CTSL and Bcl-2 in SK-N-BE (2) cells (red bar: 10 μm). NS: not statistically significant, *, *p* < 0.05, **, *p* < 0.01, ***, *p* < 0.001.

At present, it is widely accepted that anti-apoptosis is a potent inducer of chemotherapy failure. To further characterise the mechanism of underlying chemoresistance in NB, we evaluated apoptotic response with flow cytometry in SK-N-BE (2) cells with different treatments. Apoptosis levels did not significantly increase in si-CTSL (450) group compared with NC group (Supplementary Figure S5A); by contrast, a remarkable increase in the number of late apoptotic cells was observed in si-CTSL (450) plus ADM group compared with ADM group (*p* < 0.001). Then, the expression levels of Bcl-2 protein and Bax protein were detected by western blot to determine whether knockdown of CTSL could induce cell apoptosis. We found that the expression level of anti-apoptotic protein Bcl-2 in si-CTSL group under the action of ADM and DDP with the same concentrations was lower than that of NC group, and the expression level of apoptosis-related protein Bax in si-CTSL group under the action of ADM and DDP with the same concentrations was higher than that of NC group (Figure 5A; Supplementary Figures S5B,C), which indicated that knocking down CTSL induced apoptosis of SK-N-BE (2) cells. Then, we found that the fluorescence intensity of Bcl-2 protein in si-CTSL group was darker and the area was smaller than that in NC group under the action of ADM and DDP with the same concentrations using laser confocal microscope (Figure 5B). These results suggested that CTSL down-regulated ADM and DDP sensitivity in NB cells by inhibiting cell apoptosis.

### Cathepsin L induced chemoresistance by up-regulating the expression of serglycin in NB cells

**FIGURE 6:**
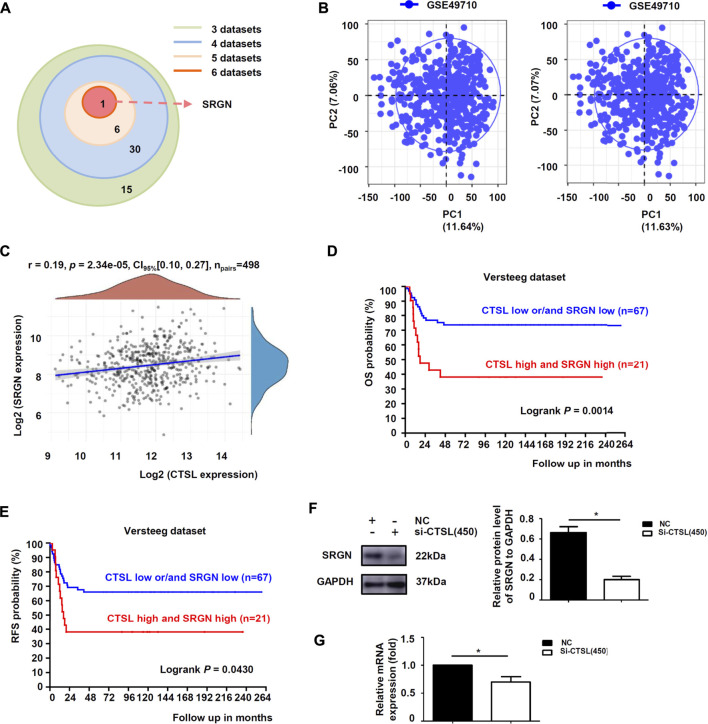
CTSL induced chemoresistance by up-regulating the expression of SRGN in NB cells **(A)** Overlap among the sets of positively-correlated genes of CTSL in NB identified by the six datasets (Tumor Neuroblastoma—Seeger—117—DCHIP; Westermann—144—tpm; Fischer—223—custom; Jagannathan—100—custom; Maris—101—custom; SEQC—498—custom) **(B)** Principal component analysis (PCA) result before batch removal for the dataset GSE49710 was show in the left, and PCA result after batch removal was show in the right **(C)** Correlation between CTSL and SRGN expression in 498 NB samples from GSE49710 dataset **(D)** Kaplan-Meier analysis of overall survival (OS) and **(E)** Recurrence-free survival (RFS) were determined according to CTSL/SRGN expression in 88 NB samples from Versteeg dataset. Statistical analysis was carried out using a log-rank test. Western blot analysis **(F)** and real-time quantitative PCR analysis **(G)** of SRGN levels in SK-N-BE (2) cells after CTSL silencing. *, *p* < 0.05.

**FIGURE 7:**
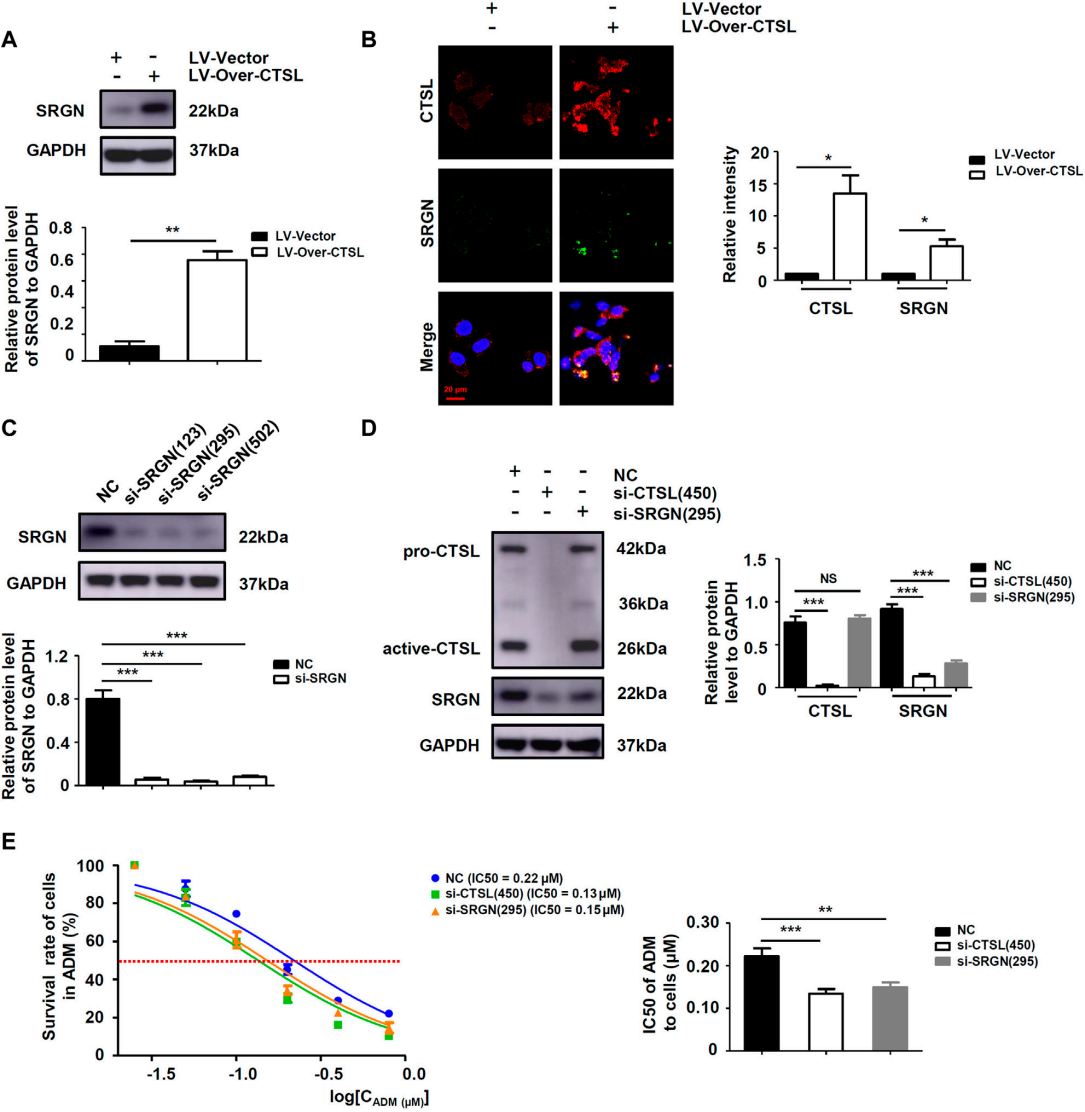
SRGN was a key protein for CTSL mediated chemoresistance in NB cells **(A)** Western blot analysis of SRGN levels in IMR-32/LV-Vector cells and IMR-32/LV-Over-CTSL cells **(B)** Immunofluorescence assay showing the expression and intracellular location of CTSL and SRGN in IMR-32/LV-Vector cells and IMR-32/LV-Over-CTSL cells. Nuclei were counterstained with DAPI (red bar: 20 μm) **(C)** and **(D)** Western blot showing SRGN expression in IMR-32/LV-Over-CTSL cells after SRGN silencing or CTSL silencing **(E)** Cell viability curves for IMR-32/LV-Over-CTSL cells after si-CTSL (450), si-SRGN (295), and ADM treatment were evaluated using the Cell Counting Kit-8 assay (left panel). The IC50 values were analyzed using the Mann-Whitney test (right panel). NS: not statistically significant, *, *p* < 0.05, **, *p* < 0.01, ***, *p* < 0.001.

To further explore the downstream targets of CTSL in NB cells, six datasets were observed at the same time and showed that only Serglycin (SRGN) expression was associated with the expression levels of CTSL (−0.6 < R < 0.6 and *p* < 0.001; Figure 6A). Preliminary data analysis and outlier identification were performed using principal component analysis (PCA). PCA results before batch removal for multiple datasets showed that the three datasets were separated without any intersection, while PCA results after batch removal showed the intersection of three datasets, which could be used as a batch of data for subsequent analysis (Figure 6B). Then Spearman’s correlation analysis showed that CTSL expression was positively correlated to SRGN expression (r: 0.19; *P*: 0.01577; 95% CI: 0.10–0.27; Figure 6C). Versteeg dataset also demonstrated that patients with high SRGN mRNA levels (cut off: 890.90) and high CTSL mRNA levels showed significantly poor OS (22-years OS; *p* = 0.0014; Figure 6D) and poor PFS (22-years OS; *p* = 0.0430; Figure 6E). Next we detected the expression levels of SRGN in si-CTSL NB cells to confirm our findings. The protein and mRNA levels of SRGN were lower in si-CTSL group than that in NC group (Figures 6F,G). To further investigate the potential links between CTSL and SRGN, we overexpressed CTSL in IMR-32 cells through lentivirus transduction. As shown in Figure 7A, CTSL overexpression increased SRGN expression in IMR-32/LV-Over-CTSL cells. Then, we found that high co-expression of CTSL with SRGN was shown in IMR-32/LV-Over-CTSL cells, as detected by immunofluorescence staining (Figure 7B). To further examine the effect of SRGN on CTSL induced chemoresistance, we suppressed SRGN by transfecting IMR-32/LV-Over-CTSL cells with three selected si-RNAs. Western blot results suggested that si-SRGN (295) inhibition evidently decreased the SRGN expression (Figure 7C). To elucidate the underlying mechanism, we performed a recovery experiment in IMR-32/LV-Over-CTSL cells which were transfected with si-SRGN (295). The results showed that the expressions of SRGN were both decreased in si-CTSL (450) group and si-SRGN (295) group compared with NC group (*p* < 0.001; Figure 7D). More importantly, we found that the IC50 of si-CTSL (450) group and si-SRGN (295) group under the treatment of ADM were significantly lower than that of NC group, which showed that the chemosensitivity of IMR-32/LV-Over-CTSL cells was mediated by CTSL-SRGN regulation (Figure 7E). All results above indicated that CTSL could mediate chemoresistance by up-regulating SRGN expression in NB cells and SRGN expression was positively correlated with poor prognosis of NB patients.

## Discussion

NB is considered as the most common pediatric solid tumor (Zafar et al., 2021). DDP and ADM are two main chemotherapeutics for NB. However, the chemoresistance of NB is the most critical reason for the failure of clinical chemotherapy (Gao and Wang, 2019). Therefore, it is important to explore the molecular mechanism that affects the chemoresistance of NB and find key targets to overcome it. Our study found that the expression level of CTSL in NB patients was positively correlated with poor prognosis and poor sensitivity of DDP and ADM. Moreover, we also found that CTSL mediated the decrease of DDP and ADM sensitivity of NB cells by up-regulating the expression of multidrug resistance proteins ABCB1 and ABCG2 and inhibiting autophagy and apoptosis. In addition, we also demonstrated that CTSL might up-regulate the expression of SRGN which was positively correlated with the poor prognosis of NB patients.

CTSL is a lysosomal enzyme, which has been demonstrated to be highly expressed in many malignant tumors such as lung cancer, breast cancer and cervical cancer (Han et al., 2016; Mao et al., 2019; Parigiani et al., 2020). CTSL plays a key role in the formation, growth, invasion and migration of malignant tumors (Sudhan and Siemann, 2015). Zhang et al. discovered that CTSL was overexpressed in ovarian cancer and CTSL could induce paclitaxel resistance in ovarian cancer cells (Zhang et al., 2016). Cui et al. also found that CTSL expression was higher in NSCLC cells and overexpression of CTSL was positively correlated with gefitinib resistance in non-small cell lung cancer (NSCLC) (Cui et al., 2016). These results indicated that CTSL might be closely related to thechemoresistance of cancers. However, the specific role and mechanism of CTSL in chemoresistance of NB have not been studied clearly. Therefore, our study mainly researched the role and molecular mechanism of CTSL in mediating chemoresistance of NB.

Firstly, we selected two different NB cells for experiments, and found that SK-N-BE (2) cells showed higher expression of CTSL and more resistant to ADM or DDP. These results indicated that CTSL might mediate the decrease of ADM and DDP sensitivity in SK-N-BE (2) cells. In order to verify the role of CTSL in mediating chemoresistance, we used CTSL targeting si-RNAs to interfere CTSL, and the chemoresistance of SK-N-BE (2) cells to ADM decreased by about 1.7 times and that of SK-N-BE (2) cells to DDP decreased by about 2.6 times. Similarly, cotreatment with CTSL inhibition and ADM in M-NSG mice of subcutaneous tumors exhibited less tumor growth. To further verify the role of CTSL in regulating the decrease of the sensitivity to ADM and DDP, we detected the expression levels of multidrug resistance proteins ABCB1 and ABCG2 by western blot and found that ABCB1 and ABCG2 expression were decreased in si-CTSL group, which indicated that the sensitivity of SK-N-BE (2) cells to ADM and DDP were significantly increased after si-RNA interfering CTSL. According to the above results, it was demonstrated that CTSL was involved in regulating the decrease of ADM and DDP sensitivity in SK-N-BE (2) cells.

To explore the mechanisms of CTSL in the chemoresistance of SK-N-BE (2) cells, the autophagy of cells was observed from the cell morphology, and the green fluorescent spots of SK-N-BE (2) cells in si-CTSL group were brighter and more, indicating that the autophagy level was higher. With autophagy protein LC3-II as an index, we observed that the autophagy level of SK-N-BE (2) cells in si-CTSL group increased significantly under the treatment of ADM and DDP. After si-RNA interfering CTSL, the expression level of pro-apoptotic protein Bax increased and the expression level of anti-apoptotic protein Bcl-2 decreased under the treatment of ADM and DDP. The above results indicated that CTSL could mediate the chemoresistance of NB by regulating the autophagy and apoptosis of SK-N-BE (2) cells.

SRGN is a low molecular weight glycoprotein, which plays a critical role in the storage and secretion of some chemokines, cytokines and proteases, thus, it participates in lots of physiological and pathological processes (Zhu et al., 2021). SRGN has been demonstrated to be overexpressed in many cancers and is closely related to the occurrence and development of tumors (Zhang et al., 2017; Xie et al., 2021; Zhu et al., 2021). Guo et al. found that SRGN was highly expressed in NSCLC and its interaction with CD44 could promote the metastasis of NSCLC (Guo et al., 2020). Moreover, Zhang et al. discovered that the crosstalk of SRGN and the transcriptional coactivator YES-associated protein mediated the chemoresistance and stemness in breast cancer cells by regulating the expression of HDAC2 (Zhang Z. et al., 2020). We here found that CTSL could elevate SRGN expression and CTSL/SRGN axis induced the chemoresistance in NB cells.

The study still has some limitations. Since this study has not constructed NB chemoresistant cells, we have not been able to clarify the role of CTSL in NB chemothresistant cells, which needed to be further verified by more experiments. Meanwhile, the mechanism about CTSL how to regulate SRGN needed to be clarified. As reported before, CTSL could get into the nucleus, and then processes and activates certain transcription factors to perform transcription functions (Sudhan and Siemann, 2015). It was reported that SRGN could be transcriptionally regulated in the tumor cells (Xu et al., 2018). Therefore, CTSL elevated SRGN expression probably through indirect endonuclear transcription fuction.

In all, our study explored the role and molecular mechanism of CTSL in regulating the chemoresistance of ADM and DDP in NB cells, and found that CTSL could mediate the chemoresistance of NB by up-regulating the expression of SRGN. Therefore, CTSL seems to play a key role in improving the chemotherapy sensitivity of NB, and it may become an important target to improve the chemosensitivity of NB and the effect of ADM and DDP.

## Data Availability

The original contributions presented in the study are included in the article/Supplementary Material, further inquiries can be directed to the corresponding authors.
