# Supplementary figures and images for: Cathepsin L promotes chemresistance to neuroblastoma by modulating serglycin
Source: Front Pharmacol. 2022 Aug 26;13:920022. doi: 10.3389/fphar.2022.920022 (PMC9484481; doi:10.3389/fphar.2022.920022)

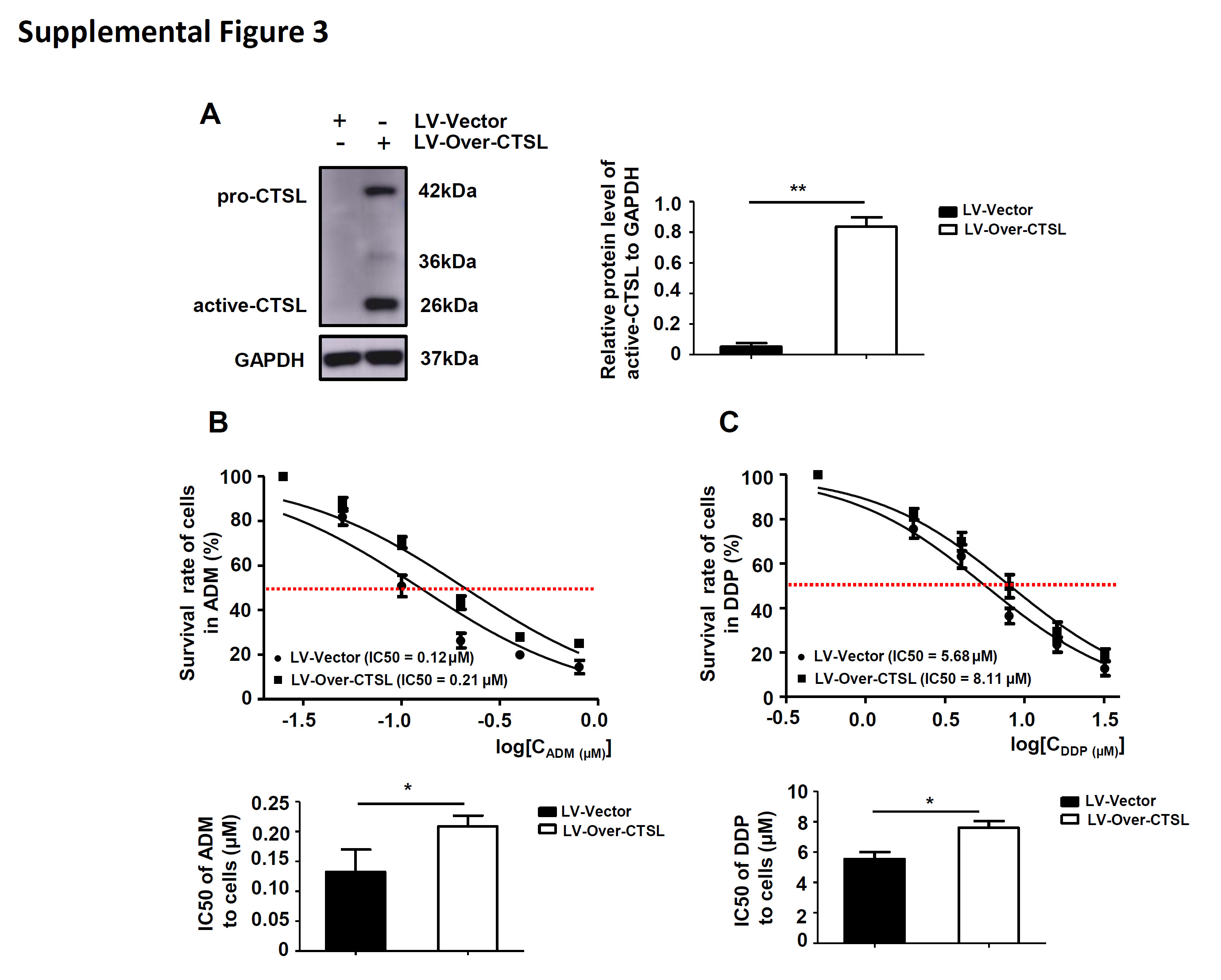

Supplement: Supplementary file 1 [file Image3.JPEG]

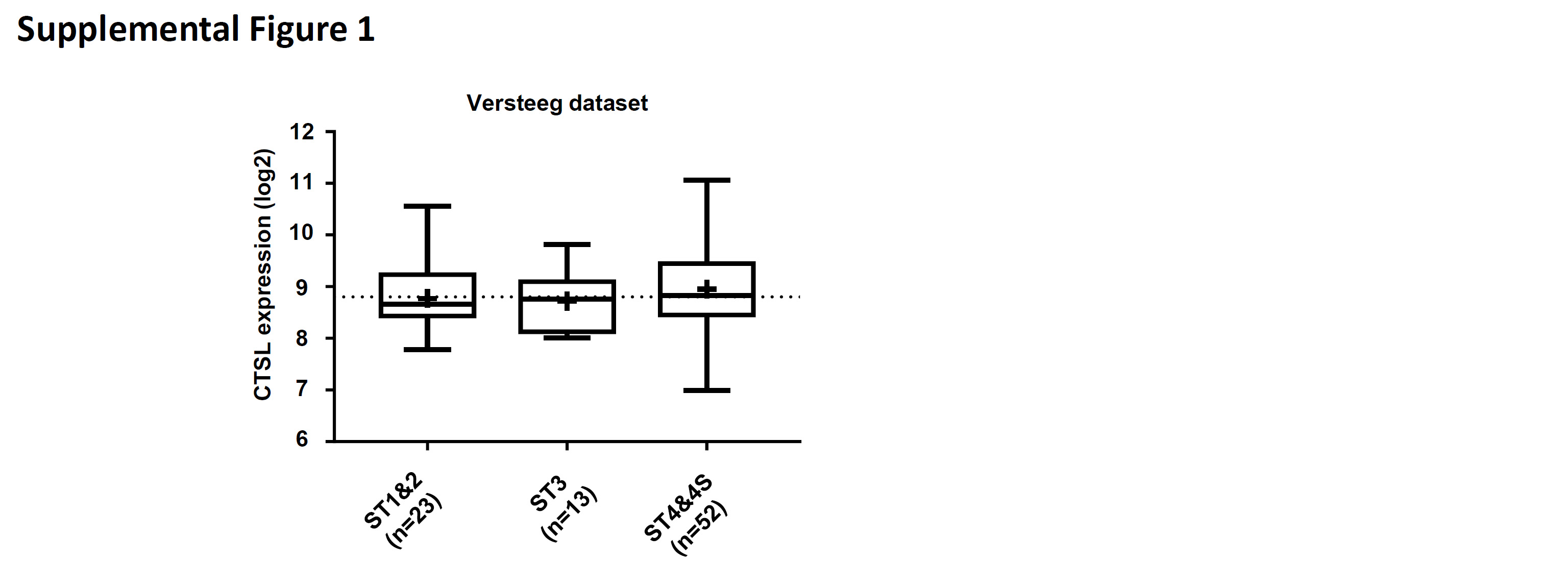

Supplement: Supplementary file 2 [file Image1.JPEG]

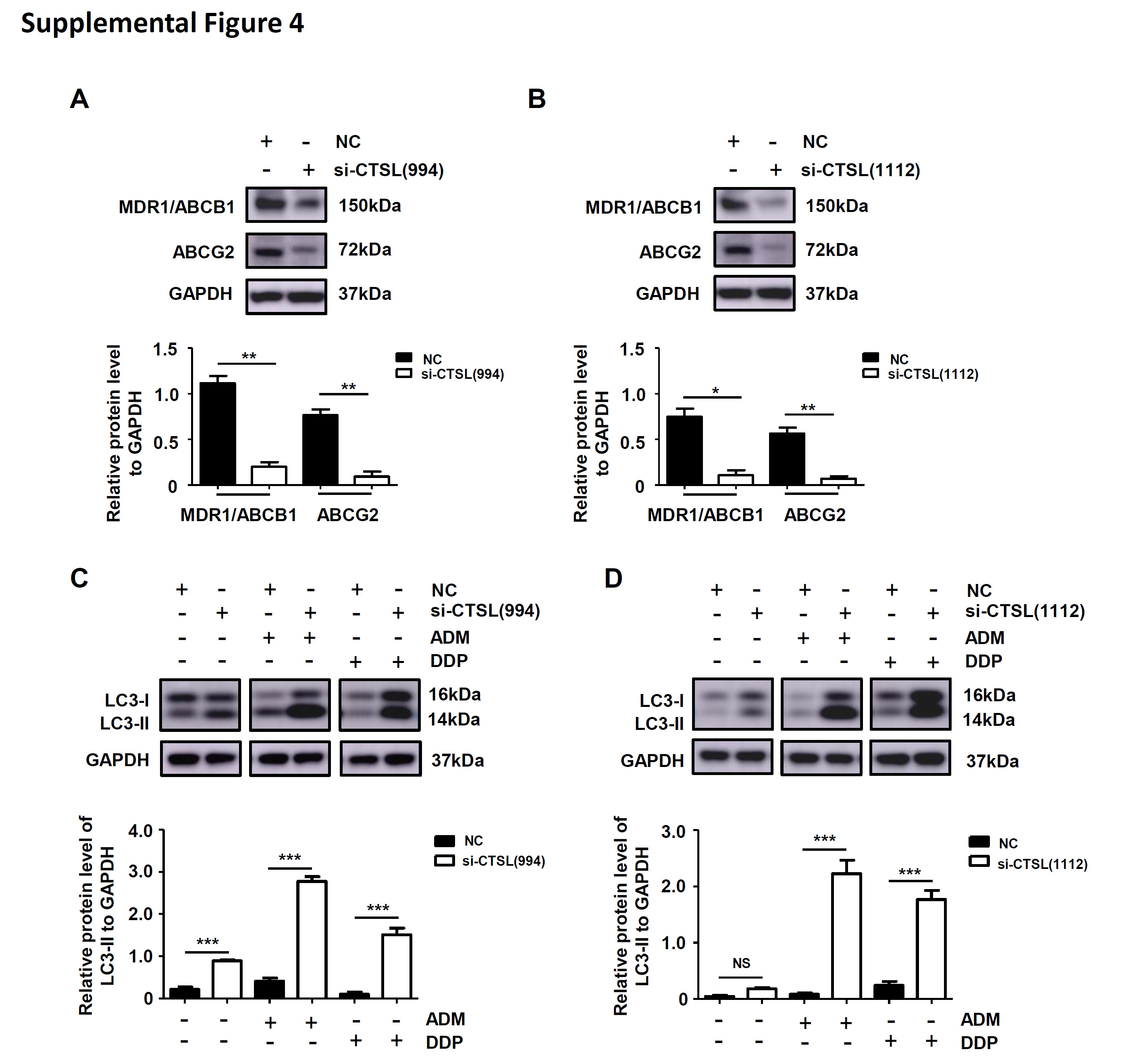

Supplement: Supplementary file 3 [file Image4.JPEG]

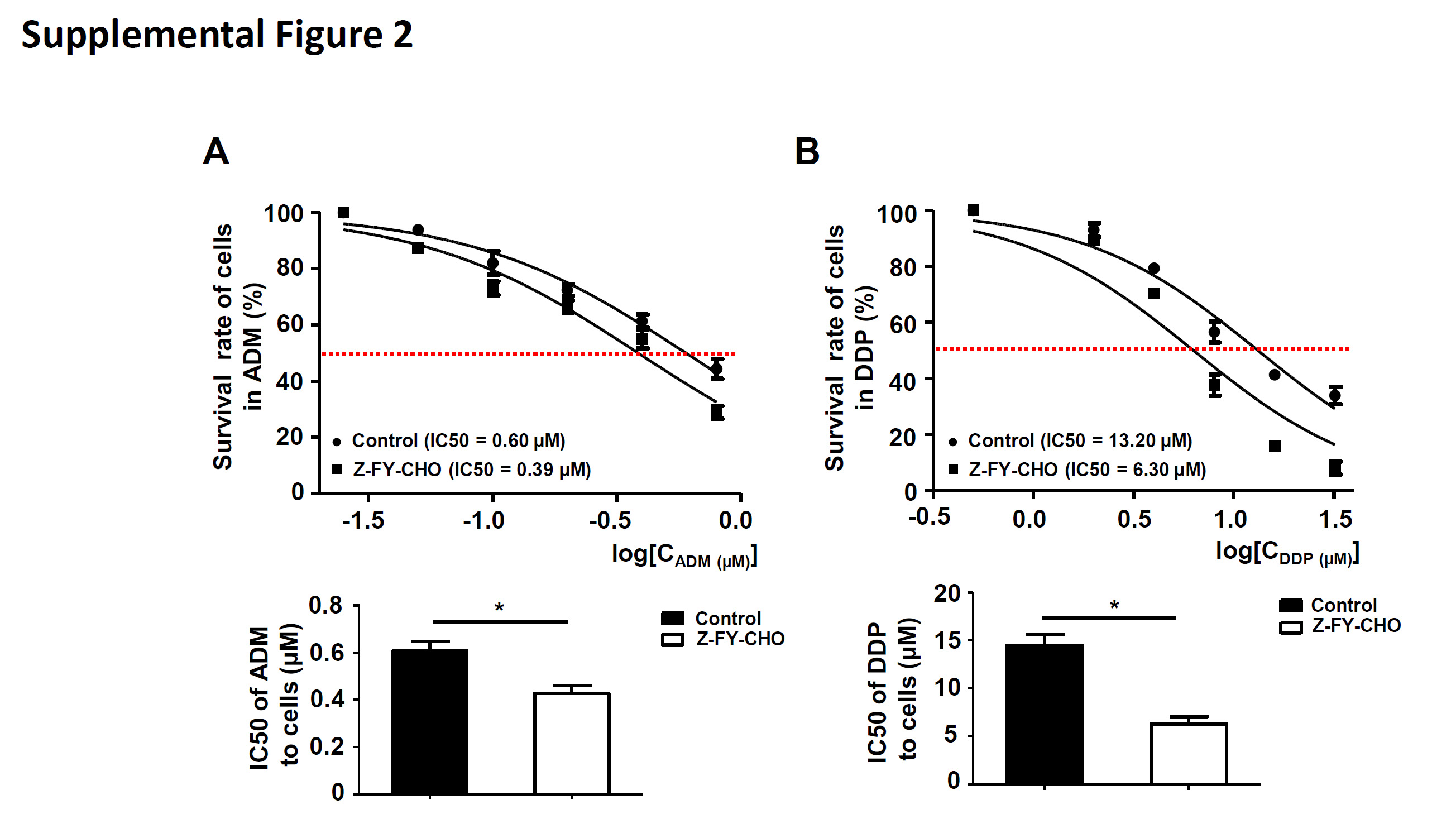

Supplement: Supplementary file 4 [file Image2.JPEG]

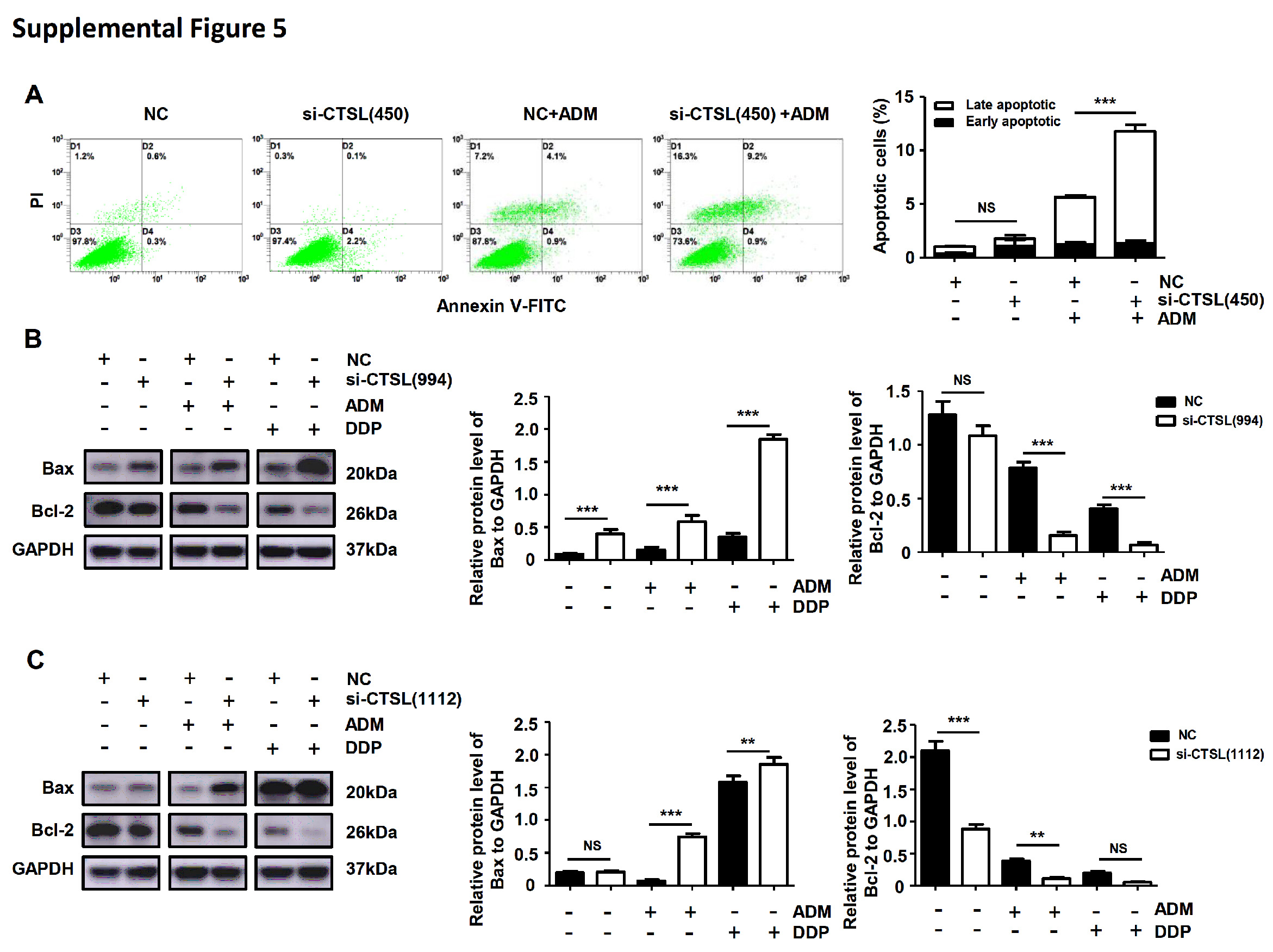

Supplement: Supplementary file 5 [file Image5.JPEG]
